# Heat, Hydration and the Human Brain, Heart and Skeletal Muscles

**DOI:** 10.1007/s40279-018-1033-y

**Published:** 2019-01-22

**Authors:** Steven J. Trangmar, José González-Alonso

**Affiliations:** 1 Department of Life Sciences, University of Roehampton, London, UK; 2 Centre for Human Performance, Exercise and Rehabilitation, College of Health and Life Sciences, Brunel University London, Uxbridge, UB8 3PH UK; 3 Division of Sport, Health and Exercise Sciences, Department of Life Sciences, College of Health and Life Sciences, Brunel University London, Uxbridge, UK

## Abstract

People undertaking prolonged vigorous exercise experience substantial bodily fluid losses due to thermoregulatory sweating. If these fluid losses are not replaced, endurance capacity may be impaired in association with a myriad of alterations in physiological function, including hyperthermia, hyperventilation, cardiovascular strain with reductions in brain, skeletal muscle and skin blood perfusion, greater reliance on muscle glycogen and cellular metabolism, alterations in neural activity and, in some conditions, compromised muscle metabolism and aerobic capacity. The physiological strain accompanying progressive exercise-induced dehydration to a level of ~ 4% of body mass loss can be attenuated or even prevented by: (1) ingesting fluids during exercise, (2) exercising in cold environments, and/or (3) working at intensities that require a small fraction of the overall body functional capacity. The impact of dehydration upon physiological function therefore depends on the functional demand evoked by exercise and environmental stress, as cardiac output, limb blood perfusion and muscle metabolism are stable or increase during small muscle mass exercise or resting conditions, but are impaired during whole-body moderate to intense exercise. Progressive dehydration is also associated with an accelerated drop in perfusion and oxygen supply to the human brain during submaximal and maximal endurance exercise. Yet their consequences on aerobic metabolism are greater in the exercising muscles because of the much smaller functional oxygen extraction reserve. This review describes how dehydration differentially impacts physiological function during exercise requiring low compared to high functional demand, with an emphasis on the responses of the human brain, heart and skeletal muscles.

## Key Points

**Table:** 

Dehydration, the process of losing body water through thermoregulatory sweating, can lead to marked alterations in physiological function and decrements in endurance performance during training and competition in temperate and hot environments.
Impaired endurance capacity in the dehydrated and hyperthermic athlete is associated with physiological strain in multiple systems, organs and tissues involving the brain, heart and skeletal muscles.
Ingesting fluids during prolonged exercise in temperate and hot environments attenuates or even prevents the deleterious effects of dehydration on physiological function and endurance performance.

## Introduction

Dehydration and hyperthermia, which are frequently experienced by humans exercising in hot environments, are major physiological stressors that can severely hinder general physiological function and cognitive and athletic performance during endurance events (i.e., long distance running, cycling or swimming, triathlon and team sports) [1–8]. The interaction among the type, duration and intensity of exercise and the environmental conditions determines the body’s overall functional demand. Dehydration poses additional stress on the human body regulatory systems, and its interaction with the overall functional demand will dictate the extent of the physiological and perceptual strain and thus whether physical performance is compromised. The endurance athlete’s training and heat acclimation status would also have an influence. In this light, the severely dehydrated, heat unacclimated and unfit endurance athlete, who is training or competing in a hot and humid environment, will likely experience the most deleterious physiological and performance effects [9, 10]. The mildly dehydrated, highly fit endurance athlete who is training and competing in a cold environment will be at the opposing end of the spectrum [10, 11].

This review considers how human physiological function is affected by exposure to heat and dehydration at rest, and during exercise of different modalities and intensities, with an emphasis on the blood perfusion and metabolism of regional tissues of the human body. The impact of dehydration and hyperthermia on physiological function will be interpreted primarily according to Ohm’s law and the Fick principle, which have blood flow as a common denominator. Ohm’s law is a fundamental law of physics that explains the physical factors that govern blood flow. When applied to the human cardiovascular system, Ohm’s law states that (driving or perfusion) pressure equals flow times resistance, with pressure being the force that drives flow and resistance the force that opposes flow. The Fick principle, in turn, determines the rate of oxygen consumption by the human body, an organ, limb or tissue. This is equal to the product of blood flow and the arterial-venous oxygen content differences. Both Ohm’s law and the Fick principle can be applied systemically to the cardiovascular system as a whole and/or regionally to, for instance, the exercising limbs, heart or brain, which are essential tissues and organs in the exercising human.

A major focus of the review is on examining the relationship between regional circulation and metabolism to evaluate whether reduced oxygen and nutrient supply to and aerobic metabolism by the human brain, heart and/or active skeletal muscle could explain the accelerated fatigue and impaired (whole-body) exercise capacity often seen in the significantly dehydrated and hyperthermic human. In this review whole-body exercise is defined as exercise that engages a large muscle mass such as cycling, running, rowing or swimming. Interested readers are directed to other topical reviews on the effects of dehydration on exercise performance [7, 12] and the interaction of possible factors contributing to fatigue during exercise in the heat [13].

## Heat, Hydration and Athletic Performance

In this section, we discuss how ambient temperature and hydration interact to influence submaximal and maximal endurance performance and aerobic capacity. The underlying physiological mechanisms are explored in different body systems in subsequent sections.

### Heat, Hydration and Submaximal Endurance Performance

Physical work capacity and endurance exercise performance are typically impaired in high ambient temperatures, particularly when accompanied by whole-body hyperthermia [4, 10, 14–19]. Galloway and Maughan, for instance, observed a ~ 45% decline in time to exhaustion when ambient air temperature was increased from 11 to 31 °C [15], a finding in agreement with other studies in the literature [15, 16, 20]. Submaximal endurance performance can be markedly degraded in the heat even with only a modest increase in core temperature [18, 21–23], suggesting a potential role of skin hyperthermia in the fatigue process. Dehydration, a natural consequence of body water losses through sweating, can compound the impairment in physical performance, but its impact is minimal when environmental temperature is low. In support of this idea, Kenefick and colleagues found that dehydration of 4% body mass reduced cycling time trial performance by up to ~ 23% in a hot environment (40 °C; no fan cooling), but the performance decrement was diminished to 12%, 5% and 3% when the ambient temperature was decreased to 30 °C, 20 °C and 10 °C, respectively [10]. The greater impact of dehydration on submaximal endurance performance in high compared to low ambient temperature is generally reflected in the available literature, where endurance performance shows little or no decrease in cooler conditions [10, 11] compared to much hotter and uncompensable environments [9, 24, 25] (see [12] for review). Interestingly, whole-body aerobic metabolism ($\dot{V}{\text{O}}_{2}$) remains at control levels during submaximal endurance exercise, despite evident physical performance degradation [26]. In addition, changes in substrate utilisation do not appear to impair aerobic exercise performance (e.g. time trial) [31] even though progressive dehydration [27–29] and hyperthermia [30] lead to enhanced carbohydrate oxidation and muscle glycogen utilisation.

### Heat, Hydration and Maximal Aerobic Capacity

While submaximal endurance performance can be markedly impaired without significant reductions in whole-body $\dot{V}{\text{O}}_{2}$, both maximal aerobic capacity ($\dot{V}{\text{O}}_{2\text{max} }$) and maximal endurance performance can be suppressed to a varied extent when performing in the very heavy and severe exercise intensity domains. Studies comprehensively exploring the independent and combined effects of body hyperthermia and dehydration on $\dot{V}{\text{O}}_{2\text{max} }$ support this idea. In some cases heat stress only minimally reduces $\dot{V}{\text{O}}_{2\text{max} }$ (≤ 3%) and endurance performance [1, 32], whereas in others, more substantial (~ 7–30%) impairments are shown [4, 5, 22, 33–38]. For example, Arngrimsson and colleagues found that $\dot{V}{\text{O}}_{2\text{max} }$ was reduced by ~ 4%, ~ 9% and ~ 18% during constant-load treadmill running at 35 °C, 40 °C and 45 °C ambient temperature, respectively. Of note, the fall in $\dot{V}{\text{O}}_{2\text{max} }$ in the highest ambient temperature (i.e. 45 °C) was halved when the same exercise was performed without any preceding warm-up, suggesting that the combination of high internal (core) and skin temperature is an important prerequisite for a reduced maximal aerobic capacity. Their study, however, did not partition the effects on $\dot{V}{\text{O}}_{2\text{max} }$ of skin hyperthermia and whole-body hyperthermia.

### Magnitude of the Circulatory Strain and Aerobic Performance

To characterise the independent effect of skin hyperthermia in conditions requiring maximal aerobic capacity, a recent study carefully manipulated body temperature with a water-perfused suit to investigate the impact of high skin temperature without an increase in core temperature on $\dot{V}{\text{O}}_{2\text{max} }$ and work capacity in endurance-trained cyclists [38]. In this study, participants performed three incremental exercise tests to volitional exhaustion with (1) skin hyperthermia (*T*_sk_ + 6 °C), (2) whole-body hyperthermia (*T*_sk_ + 6 and *T*_core_ + 1 °C) or (3) control temperatures. Whole-body hyperthermia resulted in a similar average reduction in maximal work rate (~ 13%) and $\dot{V}{\text{O}}_{2\text{max} }$ (~ 8%) to that seen in the existing literature. Importantly, skin hyperthermia alone did not compromise work or aerobic capacity, with maximal work rate and $\dot{V}{\text{O}}_{2\text{max} }$ attaining equivalent values to those seen during the control trial. In contrast to these recent findings, others have found a weak or non-existing relationship between the reduction in $\dot{V}{\text{O}}_{2\text{max} }$ and high core temperature, fuelling support for the hypothesis that high skin blood flow requirements in the heat are a predominant factor reducing maximal aerobic capacity [1, 5, 39]. This theory is based on the evidence in untrained and unacclimated individuals that the impaired $\dot{V}{\text{O}}_{2\text{max} }$ in the heat compared to a cool environment was associated with a ~ 1.2 L min^−1^ lower maximal cardiac output ($\dot{Q\ }$) [1]. By extension, the lower maximal $\dot{Q\ }$ was purported to be related to substantial elevations in skin blood flow [5]. Two observations in trained individuals argue against the skin hyperthermia hypothesis: (1) skin hyperthermia does not seem to alter general physiological function (i.e., active tissue blood flow and metabolism) compared to control conditions [38], and (2) $\dot{V}{\text{O}}_{2\text{max} }$ and maximal $\dot{Q\ }$ are not reduced below control levels [35]. Taken together, these observations indicate that, at least in trained participants, the extent of the rise in both internal and skin temperatures is an important prerequisite for the development of a considerable degree of physiological strain that results in a substantial decline in $\dot{V}{\text{O}}_{2\text{max} }$ in hot conditions.

The magnitude of whole-body hyperthermia is also an important factor in determining whether dehydration augments cardiovascular strain (i.e., reduces peripheral and systemic blood flow and mean arterial pressure and increases peripheral vascular resistance), and ultimately compromises aerobic capacity. Dehydration does not compromise or induces only small reductions in $\dot{V}{\text{O}}_{2\text{max} }$ and exercise performance in cool environments where humans experience much lower skin and core temperatures [40–42]. However, when dehydration is coupled with high ambient temperatures and therefore whole-body hyperthermia becomes apparent, $\dot{V}{\text{O}}_{2\text{max} }$ and maximal endurance performance are compromised. For example, Ganio et al. [43] discovered $\dot{V}{\text{O}}_{2\text{max} }$ to be reduced by 8.7% when participants were dehydrated by 3.7% following 120 min of submaximal cycling in the heat [43]. When the loss in body mass was minimised (e.g. by shortening the duration of submaximal exercise), or when fluid was regularly ingested, no significant fall in $\dot{V}{\text{O}}_{2\text{max} }$ was observed. Therefore, it seems that to negatively affect $\dot{V}{\text{O}}_{2\text{max} }$ and maximal endurance performance the degree of dehydration should be ≥ 3% body mass loss. A word of caution is needed here, as the physiological effects of dehydration interact with those of hyperthermia and thus the magnitude of both stressors should be considered in predicting their impact on $\dot{V}{\text{O}}_{2\text{max} }$ and maximal endurance performance.

**Fig. 1:**
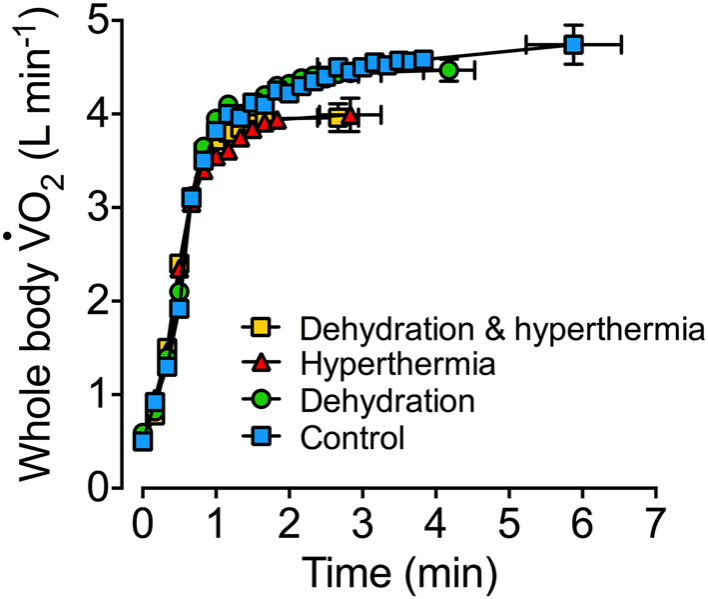
Effects of dehydration and hyperthermia on aerobic capacity and maximal endurance capacity. Oxygen consumption dynamics were measured during constant load maximal cycling (402 ± 4 W) under control, dehydration (4% body weight loss without hyperthermia), hyperthermia (+ 1 °C and + 6 °C increases in *T*_es_ and *T*_sk_, respectively) and combined dehydration and hyperthermia. Note that both combined dehydration and hyperthermia and hyperthermia alone impaired $\dot{V}{\text{O}}_{2\text{max} }$ and exercise performance by 16% and 51–53% compared to control, without altering the initial absolute $\dot{V}O_{2}$ responses. Preventing hyperthermia in dehydrated individuals restored $\dot{V}{\text{O}}_{2\text{max} }$ and exercise performance by 65% and 50%, respectively. These data demonstrate that aerobic metabolism and maximal endurance capacity can be drastically compromised in the dehydrated and hyperthermic human. Figure redrawn from data (means ± SE) reported by Nybo et al. [34]. *T*_*es*_ oesophageal temperature, *T*_*sk*_ mean skin temperature, $\dot{V}O_{2}$ oxygen uptake, $\dot{V}O_{2\text{max} }$ maximal aerobic capacity

Figure 1 illustrates the $\dot{V}{\text{O}}_{2}$ dynamics data during constant-load maximal aerobic exercise reported by Nybo and colleagues [34]. In this study, endurance-trained athletes performed four cycling bouts to exhaustion at ~ 402 W in different hydration and thermal states. Hydration status was carefully manipulated during 2 h of prior submaximal cycling in the heat to evoke either 4% body mass loss and hyperthermia (dehydration and hyperthermia), or maintain hydration status and normal body temperatures by ingesting fluid during exercise (euhydration control). In addition, thermal strain was manipulated with a water-perfused suit to induce low body temperatures during maximal exercise when dehydrated (dehydration alone) or skin and core hyperthermia when euhydrated (whole-body hyperthermia alone). The combination of dehydration and hyperthermia and whole-body hyperthermia alone reduced performance time by ~ 52% and $\dot{V}{\text{O}}_{2\text{max} }$ by ~ 16% compared to the control hydrated condition (Fig. 1). When the athletes exercised in a dehydrated state, but with low body temperatures, the falls in maximal endurance capacity and $\dot{V}{\text{O}}_{2\text{max} }$ were noticeably attenuated (~ 25% and ~ 6%, respectively). A common finding of this and previous studies is that dehydration alone can reduce maximal exercise capacity without marked reductions in $\dot{V}{\text{O}}_{2\text{max} }$ [34, 40, 41]. The earlier fatigue when dehydrated, but ‘normothermic’, likely reflects the persistent reduction in blood volume, leading to compromised locomotor and systemic blood flow and oxygen delivery, as maximal heart rate is similar at exhaustion [34, 40, 41]. In conclusion, dehydration amounting to 3–4% body mass loss reduces physical work and endurance exercise capacity and augments the development of body hyperthermia, so that these two stressors in combination can in some conditions hinder $\dot{V}{\text{O}}_{2\text{max} }$. Many of these studies, however, did not focus on the physiological mechanisms underpinning the reduced athletic performance. The following sections explore how dehydration and hyperthermia affect oxygen and the nutrient supply and metabolism of specific tissues of the human body and general physiological function.

## Heat, Hydration and the Heart

Environmental conditions and hydration status are well known physiological stressors altering central haemodynamics during submaximal and maximal exercise. Exercise in heat in the euhydrated state increases heart rate and $\dot{Q\ }$ (≥ 1 L min^−1^) and is associated with a lower arterial pressure compared to exercise in the cold [17]. The effects of exercise-induced dehydration on central haemodynamics are also magnified in the heat [17]. Cardiac output and, to a lesser extent, arterial pressure decline with graded levels of dehydration during exercise in the heat, but they remain stable or elevated during exercise in the cold [17]. Hence, the effects of environmental heat stress and dehydration interact to magnify the magnitude of physiological strain in conditions of limited evaporative cooling. Hyperthermia is a common feature of exercise-induced dehydration in the heat, whereas in the cold the same level of dehydration produces lower thermal, cardiovascular, metabolic and perceptual strain. Therefore, the greatest haemodynamic disturbances are seen during exercise in the heat when progressive dehydration and hyperthermia are combined.

**Fig. 2:**
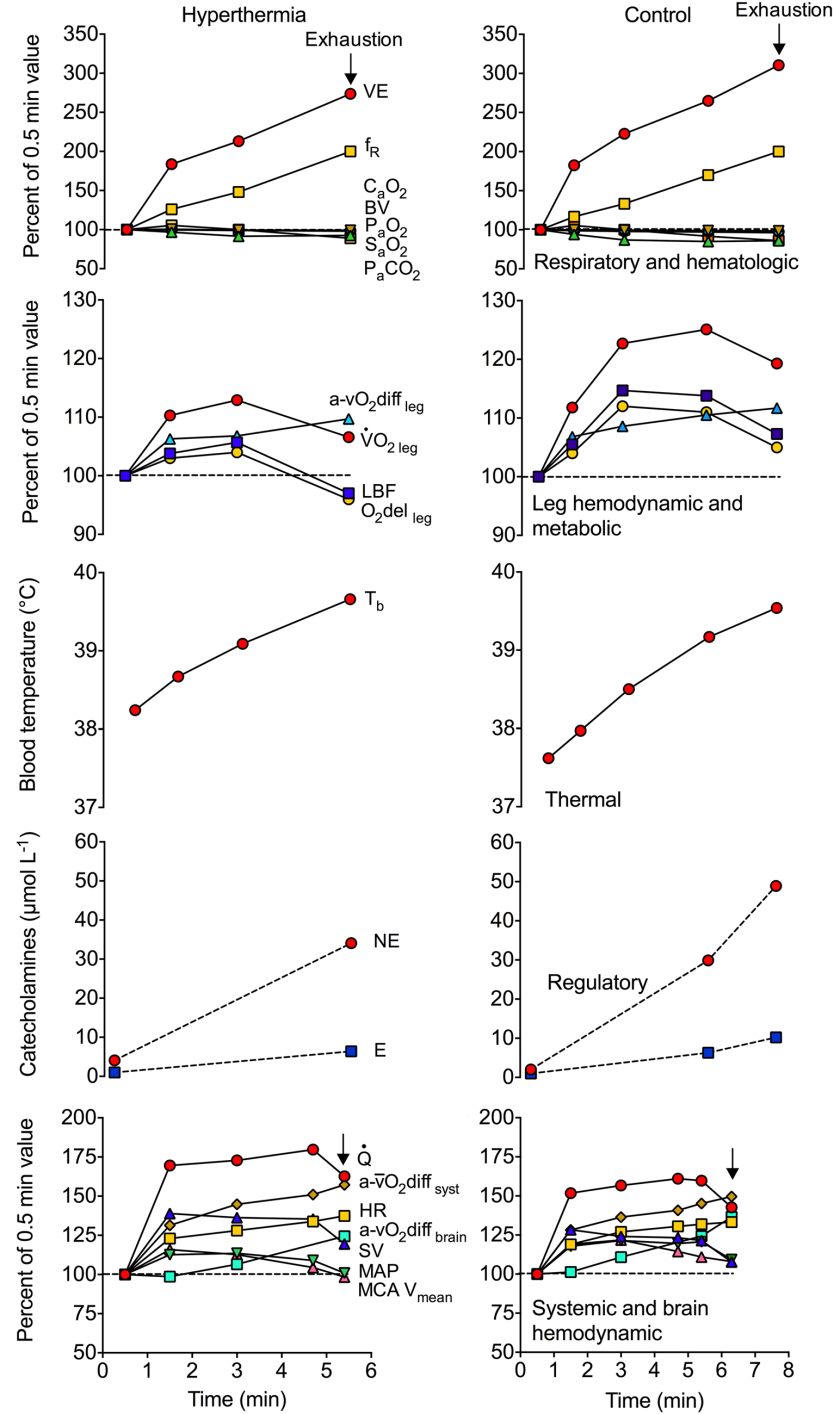
Effects of hyperthermia on respiratory, haematological, regional cardiovascular, metabolic, thermal and regulatory responses to maximal aerobic exercise and maximal endurance capacity. The over-time physiological responses are reported as a percentage of the 0.5-min exercise value, or in the case of the thermal and catecholamine responses, the delta increase in locomotor limb blood temperature and the absolute concentration values, respectively. Thermal strain was higher at the onset of constant load cycling (~ 360 W) in the whole body hyperthermia condition (+ 1 °C and 10 °C higher in *T*_es_ and *T*_sk_, respectively), and was associated with significant respiratory, cardiovascular, metabolic and regulatory strain after 3 min of cycling, leading to an accelerated fatigue (5.5 vs. 7.6 min). Note that the reductions in locomotor limb blood flow prior to exhaustion in both conditions led to a reduction in O_2_ delivery and a depressed exercising limb muscle $\dot{V}O_{2}$, even though O_2_ extraction increased. Therefore, the faster fatigue with hyperthermia and dehydration during exercise requiring aerobic capacity seems to be closely coupled with impaired muscle metabolism. Drawn from mean data reported by González-Alonso and Calbet [35] and González-Alonso et al. [107]. *VE* ventilation, *f*_*R*_ respiratory rate, *C*_a_*O*_*2*_ arterial oxygen content, *BV* blood volume, *P*_*a*_*O*_*2*_ arterial oxygen partial pressure, *S*_*a*_*O*_*2*_ arterial oxygen saturation, *P*_*a*_*CO*_*2*_ arterial carbon dioxide partial pressure, *a-vO*_*2*_*diff*_*leg*_ arterio-venous oxygen content differences across the leg, $\dot{V}O_{2 \,leg}$ leg oxygen uptake, *LBF* leg blood flow, *O*_*2*_*del*_*leg*_ oxygen delivery to the leg, *T*_*b*_ blood (femoral venous) temperature, *NE* norepinephrine, *E* epinephrine, $\dot{Q\ }$ cardiac output, *a-vO*_*2*_*diff*_*syst*_ systemic arterio-mixed venous oxygen content differences, *HR* heart rate, *a-vO*_*2*_*diff*_*brain*_ arterio-mixed venous oxygen content differences across the brain, *SV* stroke volume, *MAP* mean arterial pressure, *MCA V*_*mean*_ middle cerebral artery mean blood flow velocity

A progressive fall in $\dot{Q\ }$ is a key hallmark of the dehydration-induced cardiovascular strain observed during prolonged, strenuous whole-body exercise in the heat (Fig. 2) [44–46] and thermoneutral environments [47]. This response is prevented in trained partially heat-acclimated individuals by maintaining euhydration via ingestion of fluid during exercise [26, 45, 47, 48]. Concomitant with the declining $\dot{Q\ }$, heart rate rises continuously, whereas stroke volume declines by ~ 30% [48, 49], secondary to the rise in core body temperature and heart rate and the loss in blood volume likely limiting ventricular filling [48, 50]. Comparable core hyperthermia alone does not reduce stroke volume to the same extent (7–8%), and a number of studies show that $\dot{Q\ }$ during hyperthermic submaximal exercise is elevated, rather than reduced [2, 51–53]. These contrasting findings suggest that compromised $\dot{Q\ }$ is dependent on the extent of the cardiovascular challenge induced by combined stress evoked by dehydration, hyperthermia and exercise, or, more specifically, the overall functional demand, which is inversely related to the body functional reserve or capacity to adjust to homeostatic alterations exemplified in this case by circulatory adjustments to stroke volume reductions. To provide support for this notion, our laboratory has explored the impact of progressive dehydration on central haemodynamics at rest and during exercise of a low physiological load (i.e., single leg knee-extensor exercise) [54]. Progressive dehydration reduced blood volume (~ 5%) and lowered stroke volume (~ 20 mL) to a similar degree to that seen in the whole-body exercise paradigm, with the fall in stroke volume coupled to a substantial fall in end-diastolic volume (EDV), with only a small fall in end-systolic volume (ESV) and a marked elevation in heart rate (~ 30 bpm) compared to control (hydrated) values. Intriguingly, however, and in contrast to whole-body exercise, $\dot{Q\ }$ was maintained at rest and during single leg knee-extensor exercise across all the hydration manipulations. It therefore appears that the extent of the total muscle mass recruited, and by extension the physiological demands of the exercise, plays an important role in determining the cardiovascular strain severity induced by dehydration and hyperthermia.

### Peripheral and Cardiac Factors Reducing Stroke Volume with Dehydration and Hyperthermia

Classically, stroke volume is thought to be determined by intrinsic factors within the heart itself (i.e., cardiac mechanics, contractility and filling time) and extrinsic factors associated with alterations in preload (i.e., venous return) and afterload (i.e., arterial blood pressure and peripheral vascular resistance). The contribution of these intrinsic and extrinsic mechanisms to the dehydration-induced stroke volume reduction are discussed below, starting with the intrinsic cardiac factors. Firstly, it seems that at rest and during exercise of a low cardiovascular demand, when $\dot{Q\ }$, mean arterial pressure, systemic vascular resistance, cardiac ejection fraction and left ventricle (LV) mechanics are stable, the reduction in stroke volume is unrelated to changes in LV function [54]. Measures of systolic twist and basal rotation velocity (e.g. LV mechanics) are largely maintained or somewhat enhanced by dehydration [54], similar to findings during intermittent, submaximal exercise [55]. We cannot rule out the possibility that more substantial body mass losses in combination with more prolonged, high-intensity exercise negatively affect LV function and contribute to large stroke volume decline. In this light, some evidence suggests a depressed regional strain, torsion and untwisting velocity following an ultra-endurance activity inducing a body mass loss of ~ 4.5% [56]. However, the observation that $\dot{Q\ }$ is elevated following endurance exercise indicates that cardiac performance overall is not impaired during recovery. Moreover, the lack of effect of dehydration at rest and with low-intensity exercise [54] lends support to the argument that altered LV mechanics do not contribute to the stroke volume reduction with dehydration. Secondly, the fall in ESV with progressive dehydration at rest and small muscle mass exercise is indicative of an enhanced rather than a depressed cardiac contractility [54]. Thirdly, the progressive reduction in cardiac filling time secondary to the increase in heart rate could contribute to the reduction in stroke volume with dehydration, as seen during prolonged submaximal exercise in the heat [50, 57, 58]. The increase in heart rate is a response to reduced blood volume, and elevations in core temperature and sympathoadrenal activity [48, 59]. The contribution of cardiac tachycardia to the reduced stroke volume with dehydration is backed by the observations that (1) raising heart rate, by right atrial pacing, leads to reductions in stroke volume at any given exercise intensity [60], and (2) use of β1-adrenergic blockade during prolonged exercise prevents both the normal increase in heart rate and the normal reduction in stroke volume, probably because of the relatively longer diastolic filling time [50, 57]. Hence intrinsic factors such as cardiac contractility and mechanics do not seem to be the mechanisms explaining the stroke volume decline with dehydration and hyperthermia, but the reduction in filling time accompanying the ensuing tachycardia is an important factor.

On the other hand, the role of extrinsic factors in the stroke volume decline can be examined by scrutinising the mean arterial pressure and LV volume responses. Augmented afterload is not a factor as progressive dehydration lowers arterial pressure [48]. Instead, the diminished LV end-diastolic volume and lowered femoral beat volume indicate that venous return is compromised in conditions of dehydration and hyperthermia [26, 54]. The lower circulating blood volume resulting from the water losses via sweat and the enhanced peripheral vasoconstriction are two factors associated with the lesser venous return and left ventricular filling. These observations challenge the idea that the decline in $\dot{Q\ }$ causes the reduction in peripheral blood flow. On the contrary, it seems based on the responses of the different components of Ohm’s law that peripheral mechanisms restricting blood flow and thus venous return to the heart might account for most of the $\dot{Q\ }$ decline with dehydration and hyperthermia [60] (see further discussion in section 4). Although this hypothesis warrants further investigation, the evidence reviewed here disputes a dominant cardiac limitation, and instead suggests that the cardiac stroke volume decline with dehydration and hyperthermia at rest and during exercise is to a large extent the result of the reduced ventricular filling owing to lower venous return and filling time.

## Heat, Hydration and the Skeletal Muscles

**Fig. 3:**
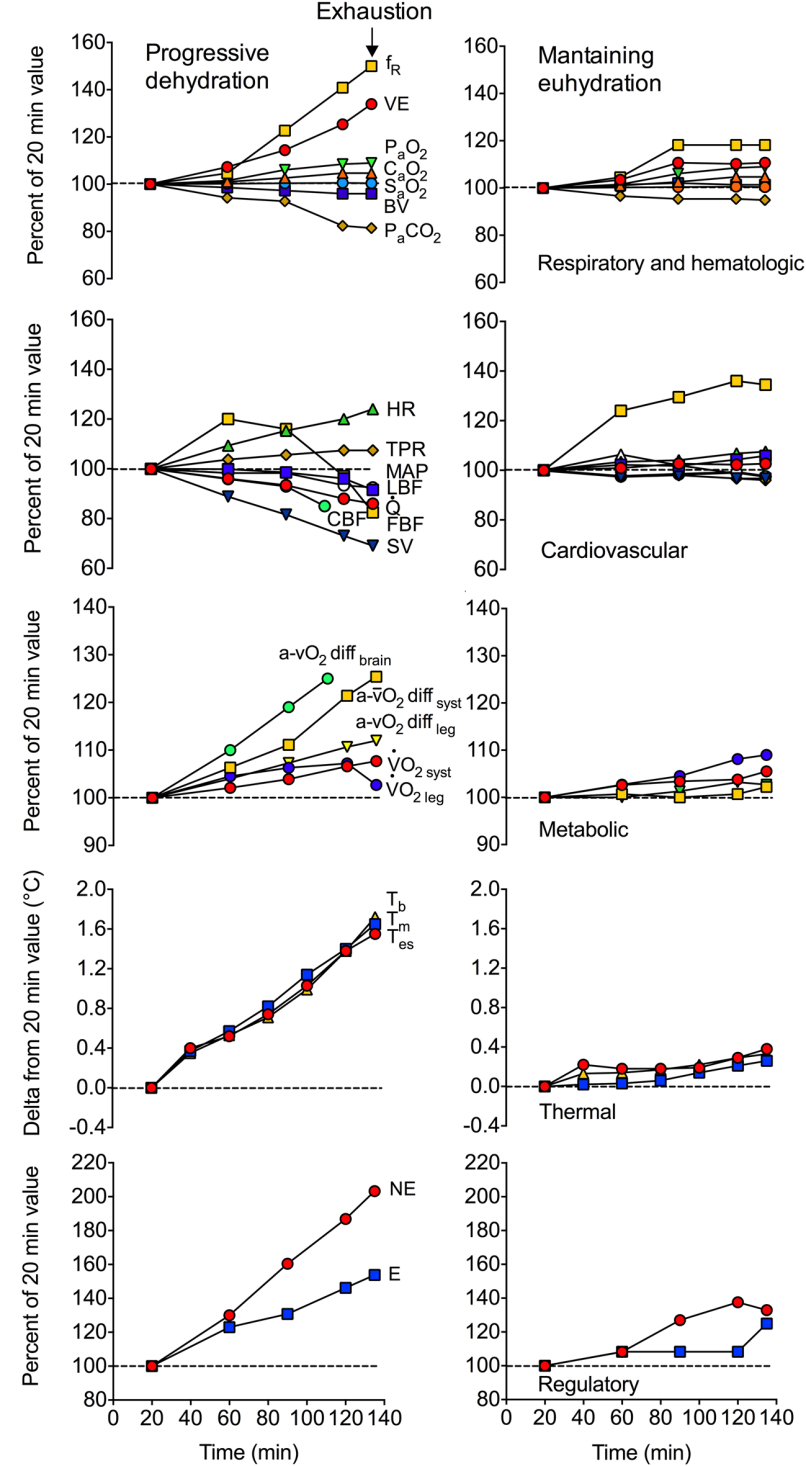
Effects of progressive dehydration and maintenance of euhydration by fluid ingestion on respiratory, hematologic, cardiovascular, metabolic, thermal and regulatory responses to prolonged exercise and submaximal endurance capacity in the heat. The over-time physiological responses are reported as percentage of the 20-min exercise value, or in the case of the thermal responses the delta increase in regional temperature. The physiological responses were not different between trials at this reference time point, as participants’ hydration status was the same. Note, however, that progressive dehydration was associated with significant physiological and perceptual strain preceding exhaustion, as reflected by gradual hyperventilation, haemoconcentration, increased arterial oxygen content, arterial hypocapnia, cardiovascular strain with reductions in brain, contracting skeletal muscle and skin perfusion, cardiac output and perfusion pressure, core and active muscle hyperthermia, alterations in neural activity, increases in perception of effort, but to a large extent maintained exercising limb, brain and systemic aerobic metabolism owing to the corresponding increases in leg, brain and systemic in oxygen extraction. This contrasts with the apparent maintenance of physiological homeostasis and perception of effort during the euhydration trial, in which athletes could have continued cycling for some additional 15–60 min before reaching exhaustion. Drawn from mean data reported by González-Alonso et al. [26, 137]. The cerebral blood flow responses were drawn from data reported by Trangmar et al. [106]. *f*_*R*_ respiratory frequency, *VE* ventilation, *P*_*a*_*O*_*2*_ arterial oxygen tension, *P*_*a*_*CO*_*2*_ arterial carbon dioxide tension, *C*_*a*_*O*_*2*_ arterial oxygen content, *S*_*a*_*O*_*2*_ arterial oxygen saturation, *BV* blood volume, *HR* heart rate, *SV* stroke volume, $\dot{Q\ }$ cardiac output, *MAP* mean arterial pressure, *LBF* leg blood flow, *TPR* total peripheral resistance, *FBF* forearm blood flow, *CBF* cerebral blood flow, *a-vO*_*2*_*diff*_*brain*_ arterio-venous oxygen content differences across the brain, *a-vO*_*2*_*diff*_*syst*_ systemic arterio-mixed venous oxygen content differences, *a-vO*_*2*_*diff*_*leg*_ arterio-venous oxygen content differences across the exercising leg, $\dot{V}O_{2 \,syst}$ systemic oxygen uptake $\dot{V}O_{2 \,leg}$ leg oxygen uptake, *T*_*b*_ blood (femoral) temperature, *T*_*m*_ muscle (vastus lateralis) temperature, *T*_*es*_ oesophageal temperature, *NE* norepinephrine, *E* epinephrine

The skeletal muscle circulation mirrors the dehydration-induced central haemodynamic alterations, at rest and during isolated-limb and strenuous whole-body exercise. At rest, limb blood flow and limb vascular conductance (limb blood flow/limb perfusion pressure) are enhanced with progressive dehydration, a response maintained during small muscle mass exercise, despite substantial body mass losses (~ 3.5%) and reductions in limb perfusion pressure [61]. Conversely, during prolonged, whole-body exercise, the substantial fall in stroke volume (47 mL beat^−1^) and $\dot{Q\ }$ (~ 4 L min^−1^) is associated with a ~ 2 L min^−1^ reduction in locomotor limb blood flow compared to control exercise [26] (see Fig. 3, left-hand panel). The marked fall in active limb blood perfusion during whole-body exercise, despite marked body hyperthermia, is offset when fluid intake matches fluid loss [26, 62], and when systemic haemodynamics are normalised (compared to upright control conditions) by exercising in a supine or semi-recumbent position [14, 63]. This close coupling between exercising limb and systemic haemodynamics is not surprising in light of the observations that pharmacologically-induced limb vasoconstriction (via intra-arterial infusion of adenosine and the sympathomimetic agent tyramine, or the combined blockade of prostaglandins and nitric oxide using NG-monomethyl-l-arginine and indomethacin infusion) decreases $\dot{Q\ }$ in proportion to the decrease in limb blood flow [64, 65], whereas limb vasodilation (via intra-arterial infusion of ATP and other nucleotides) leads to a proportional increase in limb blood flow and $\dot{Q\ }$ [66, 67], a relationship also present during hyperthermia-mediated limb hyperemia [68, 69], heat stress exercise [35, 38] and alterations in erythrocyte count and oxygenation state of haemoglobin [67]. Taken together, the exercising limb and systemic haemodynamic responses to dehydration are intimately linked, such that active skeletal muscle vasoconstriction may lead to compromised $\dot{Q\ }$ during whole body exercise at least in part by inducing reductions in venous return, left ventricular filling and ultimately stroke volume.

### Mechanisms of Blood Flow Control during Dehydration and Hyperthermia

Several local and central regulatory mechanisms may explain these differential limb haemodynamic responses to dehydration under resting and different exercise conditions. The limb blood flow responses to small muscle mass and whole-body exercise with varied hydration status can be interpreted using Ohm’s law, as the net consequence of alterations in either perfusion pressure, local vascular conductance or both. The main determinant of limb vascular conductance, the inverse of resistance, is the change in diameter of the resistance arterioles located in the muscle microcirculation. A decrease in limb vascular conductance is indicative of vasoconstriction (decreased vessel diameter), whereas an increase indicates vasodilation (increased vessel diameter). In this construct, the small increase in limb blood flow seen in small muscle mass exercise must be due to net local vasodilation, as mean arterial and limb perfusion pressure were slightly but significantly suppressed [61]. The interplay between locally released vasodilator factors and sympathetic vasoconstrictor activity primarily regulates active muscle blood flow [70–72]. It is possible that thermal, fluid and oxygen-sensing mechanisms activated by (1) increases in local tissue temperature similar to that observed during local and whole-body passive heating [68, 69, 73–75], (2) reductions in cellular volume, and (3) elevations in arterial oxygen content concomitant with the dehydration-mediated haemoconcentration [61, 76] together lead to augmented vasodilator activity in the face of a very low systemic sympathetic activity (e.g. circulating noradrenaline 1.2–1.9 nmol L^−1^). This contrasts with the responses to whole body prolonged exercise where a similar fall in mean arterial pressure (~ 8%) is accompanied by a much larger increase in circulating catecholamines (~ 18 nmol L^−1^). Paradoxically, however, vascular conductance is essentially unchanged or slightly enhanced [26, 61]. This observation is similar to those seen during constant-load, maximal exercise with superimposed body hyperthermia [35]. Taken as a whole, these findings (based on whole limb vascular conductance data) suggest that there is little or no role for actual vasoconstriction in the reduced exercising muscle blood flow with dehydration and hyperthermia during whole body exercise, but rather this is a passive event caused by the overall circulatory strain and manifested in conditions of high physiological load. Regardless of the mechanism, the large fall in active-muscle perfusion during strenuous whole-body exercise can reduce O_2_ supply, which is initially compensated by increases in oxygen extraction, but in conditions requiring near or maximal aerobic capacity would eventually lead to compromised local and systemic aerobic metabolism and accelerated fatigue, as discussed in section 6 [34, 35].

## Heat, Hydration and the Brain

Despite its relatively small contribution to total body weight, the human brain is a highly metabolically active organ, accounting for ~ 20% of whole-body aerobic metabolism and ~ 15% (750 mL min^−1^ or ~ 52 mL 100 g^−1^ min^−1^) of the resting $\dot{Q\ }$ [77–79]. The brain does not have the capacity to store oxygen and so the maintenance of its high metabolic demand is dependent on the provision of oxygen, and other metabolic substrates, and removal of metabolic by-products, by the circulation. How exhaustive exercise and stressors such as hyperthermia and dehydration interact to affect the human brain circulation and metabolism has been a major focus of research in the last two decades. We first discuss how cerebral blood flow (CBF) is altered in response to an exercise stimulus, before delving into the impact of hyperthermia and dehydration, and their effect on cerebral metabolism.

In the rest-to-exercise transition, CBF was originally shown to remain unchanged [80–85]. These investigations, however, used the Kety-Schmidt method, which determines global (i.e., whole-brain) CBF by first saturating the cerebral tissue with an inert gas (e.g. nitrous oxide [N_2_O]), before determining the ratio between the rate of N_2_O uptake and the arterial to internal jugular venous N_2_O difference by the application of the Fick principle. There are limitations that can undermine these measurements during whole-body strenuous exercise including: (1) the requirement for exercise to be of sufficient duration (~ 15 min) and of a steady-state nature (not the case during high or severe intensity exercise domains) [86], and (2) the observation that the main sampling site for obtaining venous blood drained from the brain (the internal-jugular vein) is temporally collapsed during upright exercise [87, 88], thus leading to heterogeneity of the venous drainage with the potential to miss the important metabolic profile of the brain [89]. More recent methodological approaches with good temporal resolution consistently show an exercise-induced increase in CBF by ~ 20% up to moderate exercise intensities [87, 90–101]. Thereafter, CBF remains elevated throughout the duration of prolonged exercise in the moderate-intensity domain (e.g. ≤ ~ 60% $\dot{V}{\text{O}}_{2\text{max} }$) [100], but CBF is markedly suppressed when exercise intensity is increased, often returning to near resting values prior to exhaustion [38, 87, 94, 95, 97].

**Fig. 4:**
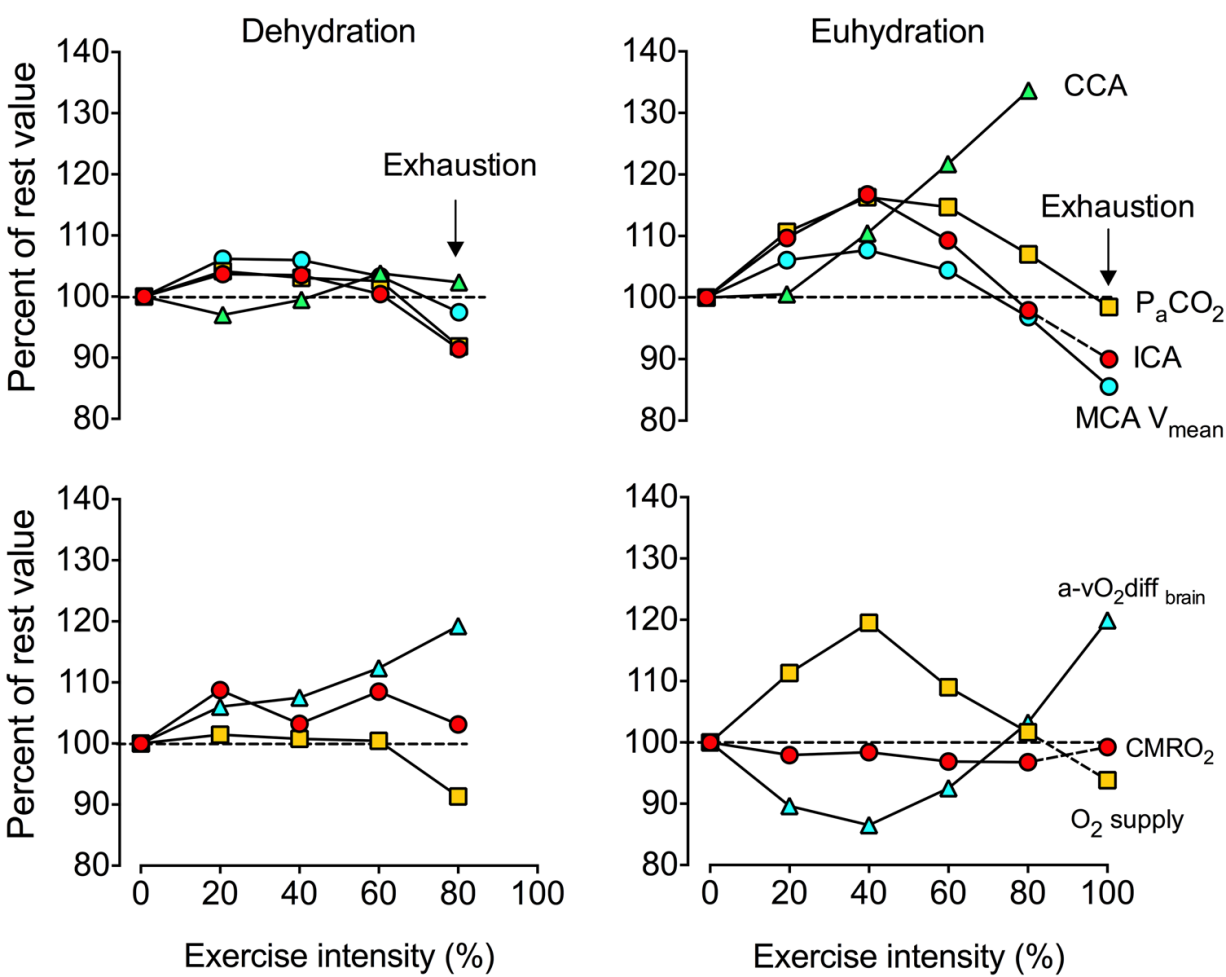
Effects of dehydration on cerebral haemodynamics and metabolism. Cerebral haemodynamic and metabolic responses during incremental cycling to exhaustion reported as percent rest reference value. Of note are the declines in cerebral blood flow and oxygen supply at about 60% of maximal aerobic exercise intensity, which are paralleled by proportional increases in oxygen extraction across the brain, such that cerebral aerobic metabolism is maintained. Drawn from mean data reported by Trangmar et al. [108]. *CCA* common carotid artery blood flow, *ICA* internal carotid blood flow, *P*_*a*_*CO*_*2*_ arterial carbon dioxide partial pressure, *MCA V*_*mean*_ middle cerebral artery mean blood flow velocity, *a-vO*_*2*_*diff*_*brain*_ differences in oxygen content across the brain, *O*_*2*_*supply* oxygen delivery to the brain which is the product of cerebral blood flow and arterial oxygen content, *CMRO*_*2*_ cerebral metabolic rate for oxygen

At rest, elevations in core temperature (e.g. by + 1.5 °C) reduce CBF by ~ 15% [102], whereas dehydration without concomitant hyperthermia elevates CBF [103]. Hyperthermia and dehydration also alter the CBF dynamics during different types of exercise. For example, CBF (MCA *V*_mean_; middle cerebral artery mean blood flow velocity) is suppressed throughout the duration of self-paced time trial, or markedly reduced by ~ 15–25% when hyperthermia develops in an uncompensable environment compared to a cool or thermoneutral environment [100, 104, 105]. The development of dehydration (≥ 3% body mass loss) during prolonged exercise in the heat causes further cerebrovascular instability by hastening the decline in CBF, concomitant with an elevated hyperthermia, tachycardia and early fatigue [106]. On the other hand, the reduction in CBF is attenuated when fluid intake matches bodily fluids lost through sweating. The effect of hyperthermia and dehydration on CBF is not isolated to prolonged exercise, as the typically observed fall in CBF during severe-intensity exercise is accelerated (i.e. occurring at a lower work rate or shorter duration of exercise) in an uncompensable hot environment [38, 107]. This is also the case when dehydrated individuals perform incremental exercise in a compensable hot environment [108], where dehydration and concomitant hyperthermia reduce CBF to values equivalent to control conditions, but at a significantly lower absolute work rate (~ 270 vs. ~ 340 W) (Fig. 4). The CBF dynamics are, however, normalised to control conditions when participants maintained their normal hydration status [108]. In summary, exercise in the heat evoking severe body hyperthermia compromises cerebral perfusion during strenuous submaximal and maximal exercise. The development of dehydration enhances the cerebrovascular strain and accentuates the fall in CBF.

### Mechanisms of CBF Control during Dehydration and Hyperthermia

The compromised CBF seen during exercise in the heat, with and without dehydration, is coupled with a fall in cerebrovascular conductance [108]. The precise regulation of cerebrovascular tone is inherently complex, potentially involving many different respiratory, metabolic and neural signals [109, 110]. The combination of hyperthermia, dehydration and strenuous exercise augments circulating noradrenaline; however, the direct effect of enhanced sympathetic vasoconstrictor activity on the cerebral vasculature is as yet unclear [111]. A more likely candidate explaining the fall in CBF in the hyperthermic and dehydrated athlete is the hyperthermia-induced hyperventilation and concomitant lowering of the partial pressure of arterial CO_2_ (P_a_CO_2_) [100, 108]. Progressive reductions in P_a_CO_2_ reduce CBF in the resting human [112] and account for all of the reduction in CBF during severe passive hyperthermia [113, 114]. A reduced P_a_CO_2_ also plays a prominent role in the fall in CBF during prolonged exercise in the heat [100], and with superimposed dehydration [106]. The early fall in CBF when dehydrated is associated with a higher core temperature and hyperventilation and a greater fall in P_a_CO_2_ [108].

### Heat, Hydration and Cerebral Aerobic Metabolism

The early reductions in CBF seen during strenuous exercise in the heat, with and without dehydration, have been postulated to reduce cerebral oxygenation [115]. A lower vascular and neuronal oxygenation could potentially compromise the cerebral metabolic rate for oxygen (CMRO_2_), thereby contributing to the overall process of fatigue [115], reductions in motor output [116, 117] and reductions in cognitive performance [118–121] that are magnified in hot environments and/or when dehydrated [118, 122–124]. The characterisation of the CMRO_2_ dynamics during exhaustive exercise is therefore a key question in integrative human physiology. CMRO_2_ is calculated using the Fick principle, as CBF × the arterial-to-jugular venous O_2_ content difference (or O_2_ extraction). The available, albeit limited, data indicate that CMRO_2_ is largely unaltered from rest-to-moderate intensity exercise [82, 83, 87], before seemingly increasing close to maximal intensities [107, 115, 125, 126]. It could be anticipated that the accelerated reductions in CBF seen with dehydration and hyperthermia during exercise in the heat would be accompanied by a decrease in the CMRO_2_. However, the available evidence does not support this idea. In two studies, CMRO_2_ appears to even increase during exhaustive exercise, as the increase in cerebral O_2_ extraction was greater than the fall in CBF (up to ~ 32% vs. ~ 15–20%) [104, 107]. The increase in CMRO_2_ was postulated to be due to the substantial core hyperthermia and related Q_10_ effect, and the heightened ‘cognitive’ effort to maintain the required physiological output when exercising in the heat. Further supporting that CMRO_2_ is not compromised, we recently observed a maintained CMRO_2_ during exercise in the heat across a range of exercise intensities and hydration states (Figs. 3 and 4). However, we did not see the previously reported increase during exhaustive exercise, as the CBF reductions and the compensatory increases in cerebral O_2_ extraction were proportional. The differential CMRO_2_ responses (increased vs. maintained CMRO_2_) are likely due to methodological differences in the calculation of CMRO_2_, specifically, whether CBF is obtained globally by the Kety–Schmidt method [104], or regionally using assumed values [107]. It is likely that global CMRO_2_ is underestimated when CMRO_2_ is quantified using oxygenation values measured in the internal jugular vein, and in our case volumetric CBF was only measured in the internal carotid artery (ICA; perfusing ~ 75–80% of the brain) [106, 108], which could be somewhat different to the responses of the posterior cerebral circulation [92, 95]. Ideally, blood flow in the vertebral arteries (accounting for the remaining ~ 20% of CBF) and oxygenation in the vertebral veins should be assessed simultaneously [127]. Notwithstanding these limitations, these studies consistently show a large cerebral O_2_ extraction reserve and a maintained or enhanced CMRO_2_, even in the most strenuous of exercise conditions, and therefore a reduced global cerebral oxygen metabolism is unlikely to explain the early fatigue seen in hot conditions while in the dehydrated and/or hyperthermic state.

## Heat, Hydration and the Mechanisms of Fatigue

In the following sections, we present two mechanistic figures to attempt to explain the primary cardiovascular and metabolic consequences of combined dehydration and hyperthermia during submaximal and maximal endurance performance. Notwithstanding this focus, it is important to recognise that the development of fatigue during strenuous exercise in the heat is a complex phenomenon, possibly involving multiple signals and processes originating in different bodily tissues and organs. In this light, the early fatigue seen during both submaximal and maximal exercise could be the net result of (but not limited to) (1) respiratory, cardiovascular and metabolic strain, (2) alterations in the functioning of the central nervous system, neurotransmitter activity and central motor output, and (3) psychophysiological factors relating to motivation, perceptions of effort and thermal comfort [12, 13, 128]. Moreover, the extent of the ensuing physiological strain is also dependent on the degree of heat acclimation and aerobic fitness of participants, as these factors have a bearing on the capacity to cope with heat stress exercise [14, 45, 48]. The highest physiological strain is expected to happen in untrained and unacclimated subjects who perform strenuous exercise in a hot and humid environment. Elite high performance and heat-acclimated athletes will be on the other end of a continuum and show minimal alterations in physiological function under comparable exercise and environmental stress [129].

### Mechanisms of Performance Limitation in Submaximal Exercise

**Fig. 5:**
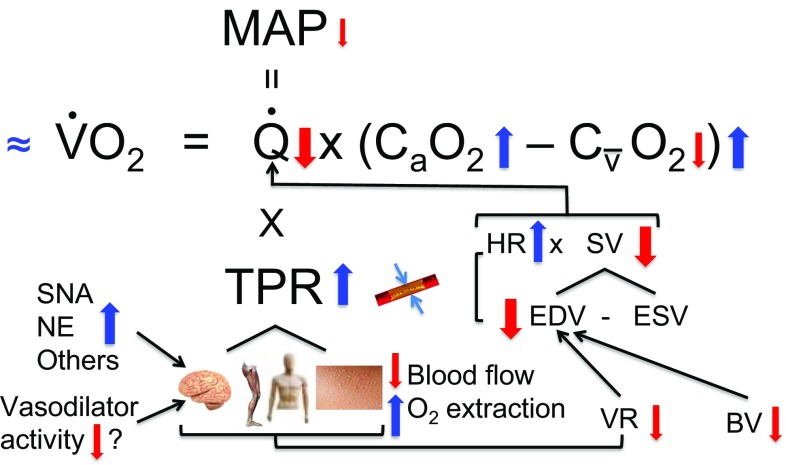
Schematic diagram illustrating the effects of dehydration and hyperthermia on physiological function during submaximal exercise, according to Ohm’s law and the Fick principle. Significant dehydration and hyperthermia (i.e., 4–5% body mass loss and 40–41 °C core and muscle temperatures) impair submaximal endurance capacity in athletes. The compromised performance occurs in association with reductions in systemic and regional tissue and organ blood flow, but compensatory physiological adjustments maintain whole-body oxygen uptake. For a full description, see section 6.1. The direction of the arrow indicates the direction of response, whereas its size indicates the magnitude of the response. $\dot{Q\ }$ cardiac output, *TPR* total peripheral resistance, *MAP* mean arterial pressure, *BV* blood volume, *VR* venous return, *SNA* sympathetic nerve activity, *NE* circulating noradrenaline concentration, *C*_*a*_*O*_*2*_ arterial oxygen content, *C*_*v*_*O*_*2*_ mixed venous oxygen content, *HR* heart rate, *SV* stroke volume, *EDV* end-diastolic volume, *ESV* end-systolic volume

The severe physiological strain induced by significant dehydration and hyperthermia is associated with the impaired submaximal endurance capacity in athletes and is typified by marked declines in blood flow to active skeletal muscle, skin and brain and $\dot{Q\ }$, a substantial increase in total peripheral resistance and a small reduction in mean arterial pressure or perfusion pressure (Fig. 5) [26, 45, 48, 49]. The reduction in $\dot{Q\ }$ is primarily related to the lowering in stroke volume, owing to the hyperthermia-induced cardiac tachycardia [48, 50, 57, 58] and concomitant reductions in blood volume and venous return [17]. The latter is related to peripheral vasoconstriction occurring in the brain, active skeletal muscle, skin and perhaps other upper body organs and tissues such as visceral organs. Lower tissue blood flow is related to the interaction between increases in vasoconstrictor activity (including sympathetic nerve activity) and alterations in vasodilator activity, although their specific contributions are as yet unknown [72]. Notwithstanding, the reductions in systemic, brain, locomotor limbs, skin and upper body tissues and organs blood flow and O_2_ supply are compensated for by increases in tissue O_2_ extraction, such that whole body $\dot{V}{\text{O}}_{2}$ is preserved [26]. The development of fatigue, when dehydrated and hyperthermic, during prolonged submaximal exercise is associated with the attainment of near to maximal heart rate and an augmented internal body temperature [45, 48, 130], the latter with implications for the central nervous system [131]. However, identifying the precise mechanism is challenging, given the multitude of circulatory, respiratory, perceptual and central processes, occurring simultaneously, that interrelate to advance fatigue [12, 13].

### Mechanisms of Performance Limitation in Maximal Exercise

**Fig. 6:**
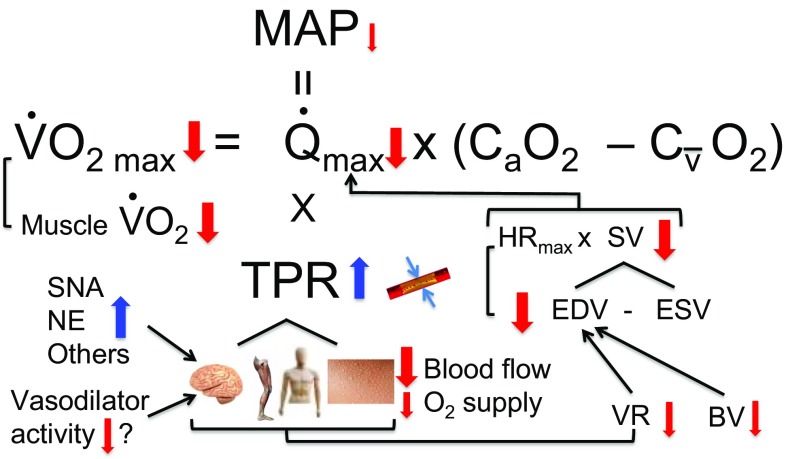
Schematic diagram highlighting major physiological factors limiting maximal endurance capacity with and without dehydration and hyperthermia. Impaired maximal endurance capacity in dehydrated and hyperthermic athletes (i.e., 4–5% body mass loss and 39–40 °C core and muscle temperatures) is associated with a marked decline in cardiac output ($\dot{Q\ }$), a substantial increase in total peripheral resistance (TPR) and a small reduction in mean arterial pressure (MAP), or perfusion pressure. The concomitant reductions in peripheral blood flow and O_2_ supply lead to suppressed whole body and locomotor limb $\dot{V}O_{2}$ because the functional oxygen extraction reserve in active skeletal muscle has been exhausted. A lower stroke volume is also the main factor reducing $\dot{Q\ }$. Peripheral vasoconstriction is proposed as a major factor reducing venous return, cardiac filling and cardiac stroke volume. The direction of the arrow indicates the direction of response, whereas its size indicates the magnitude of the response. $\dot{Q\ }$ cardiac output, *TPR* total peripheral resistance, *MAP* mean arterial pressure, *BV* blood volume, *VR* venous return, *SNA* sympathetic nerve activity, *NE* circulating noradrenaline concentration, *C*_*a*_*O*_*2*_ arterial oxygen content, *C*_*v*_*O*_*2*_ mixed venous oxygen content, *HR* heart rate, *SV* stroke volume, *EDV* end-diastolic volume, *ESV* end-systolic volume

Figure 6 outlines how the severe physiological strain induced by significant dehydration and hyperthermia impairs exercise capacity at intensities close to or at $\dot{V}{\text{O}}_{2\text{max} }$. In accordance with the Fick principle, the maximal convective O_2_ transport and O_2_ extraction (a-vO_2diff_) set the upper limit for local tissue and systemic $\dot{V}{\text{O}}_{2}$. Systemic, active skeletal muscle and brain O_2_ delivery are markedly suppressed during high-intensity exercise to volitional exhaustion [35, 95, 107, 132, 133] and, as outlined in sections 4 and 5, the early fatigue with dehydration and hyperthermia is associated with a faster decline in peripheral blood perfusion. The early attenuation in the rate of O_2_ delivery is temporally associated with the attainment of the limit of functional O_2_ extraction reserve (~ 90%) across the locomotor limbs, with the consequence that active skeletal muscle $\dot{V}{\text{O}}_{2}$ and $\dot{V}{\text{O}}_{{2{\text{max} }}}$ are blunted [35, 38]. Contrastingly, the brain does not appear to attain the limit of its functional O_2_ reserve at exhaustion (~ 40%) and, despite a marked fall in CBF, CMRO_2_ is preserved [108]. The decline in $\dot{Q\ }$ seen during maximal exercise, with and without hyperthermia and dehydration, is due to reductions in stroke volume (SV). In the context of the dehydrated and hyperthermic athlete exercising at maximal intensities, the fall in SV might be related to the reduction in EDV, owing to reductions in blood volume and the diminished venous return paralleling peripheral vasoconstriction (Fig. 6).

### Interaction of Environment and Exercise Intensity on Physiological Function

The two scenarios presented above show that the combination of dehydration, hyperthermia and exhaustive exercise leads to considerable physiological strain underpinning functional alterations in multiple organs and tissues, which directly or indirectly could curtail exercise capacity. But as discussed in Sect. 3, 4 and 5, there are numerous conditions whereby global reductions in heart, active skeletal muscle and brain O_2_ delivery and tissue metabolism do not explain the development of fatigue. For instance when exercise intensity is low, or involves a small proportion of the total muscle mass, dehydration can reduce exercise duration [134], unrelated to reduced O_2_ delivery, $\dot{V}{\text{O}}_{2}$ or the accumulation of muscle metabolites [52, 54, 61, 134]. Moreover, physiological function is largely preserved when exercise is performed in a cold environment (~ 8–10 °C) [7, 10, 17, 135], despite reductions in plasma and blood volume that might decrease stroke volume if exercise is prolonged and intense [17]. This idea is supported by evidence that fatigue also occurs when central and peripheral haemodynamics and body temperatures are stabilised during strenuous prolonged exercise in the heat, when participants match their sweat loss with a proportional intake of fluids (Fig. 3, right-hand panel) [10, 26, 48, 49]. Thus, fatigue in exercise scenarios with apparent systemic physiological stability is likely to involve a number of local tissue, cellular and molecular mechanisms that are beyond the scope of this review, but which are comprehensively discussed elsewhere [136].

## Conclusions

Submaximal and maximal endurance performance can be impaired by dehydration and body hyperthermia. The impaired physical performance is associated with a myriad of alterations in physiological function, including reduced oxygen delivery to multiple regional tissues and organs, enhanced reliance on muscle glycogen and cellular metabolism, changes in neural activity and in some exercise and environmental conditions requiring near to maximal functional capacity, compromised muscle aerobic metabolism and aerobic capacity. It is evident, however, that the level of dehydration, the intensity of the exercise and the external environmental conditions determine the extent to which general physiological function is impaired. As presented in this review, dehydration and hyperthermia can elevate blood flow to heart, active muscles and brain during low-intensity exercise. When exercise intensity is increased above moderate levels or when exercise duration is prolonged, brain, active muscle and systemic blood flow are gradually compromised, mechanistically associated with enhanced peripheral vasoconstrictor activity, suppressed venous return and cardiac filling that ultimately hinder cardiac output. This attenuation in peripheral blood flow has a different effect on regional tissue aerobic metabolism, where a compromised active muscle, but not brain, oxygen uptake is a likely precursor to early fatigue when severe intensity exercise is performed in hot environments whilst experiencing marked dehydration and hyperthermia. Future research should elucidate the contribution of extrinsic and intrinsic cardiac factors altering peripheral blood flow and cardiac output with manipulation of hydration and local and body temperatures, and explore any possible associations with the onset of fatigue during submaximal and maximal endurance exercise.
